# A mathematical model of CO_2_, O_2_ and N_2_ exchange during venovenous extracorporeal membrane oxygenation

**DOI:** 10.1186/s40635-018-0183-4

**Published:** 2018-08-09

**Authors:** Christopher John Joyce, Kiran Shekar, David Andrew Cook

**Affiliations:** 1 Discipline of Anaesthesiology Critical Care, University of Queensland, Ned Hanlon Building, Royal Brisbane and Women’s Hospital, Herston, QLD 4006 Australia; 2 Department of Intensive Care, Princess Alexandra Hospital, 199 Ipswich Rd, Woolloongabba, QLD 4102 Australia; 3 Adult Intensive Care Services, The Prince Charles Hospital, Rode Rd., Chermside, Brisbane, QLD 4032 Australia; 4 Science and Engineering Faculty, Queensland University of Technology, 2 George St, Brisbane, QLD 4000 Australia; 5 Critical Care Research Group and the Centre of Research Excellence for Advanced Cardiorespiratory Therapies Improving Organ Support (ACTIONS), Brisbane, QLD Australia

**Keywords:** Extracorporeal membrane oxygenation, ECMO, Venovenous ECMO, Extracorporeal CO_2_ removal, Gas exchange, Mathematical model, Respiratory failure, Acute respiratory distress syndrome

## Abstract

**Background:**

Venovenous extracorporeal membrane oxygenation (vv-ECMO) is an effective treatment for severe respiratory failure. The interaction between the cardiorespiratory system and the oxygenator can be explored with mathematical models. Understanding the physiology will help the clinician optimise therapy. As others have examined O_2_ exchange, the main focus of this study was on CO_2_ exchange.

**Methods:**

A model of the cardiorespiratory system during vv-ECMO was developed, incorporating O_2_, CO_2_ and N_2_ exchange in both the lung and the oxygenator. We modelled lungs with shunt fractions varying from 0 to 1, covering the plausible range from normal lung to severe acute respiratory distress syndrome. The effects on P_a_CO_2_ of varying the input parameters for the cardiorespiratory system and for the oxygenator were examined.

**Results:**

P_a_CO_2_ increased as the shunt fraction in the lung and metabolic CO_2_ production rose. Changes in haemoglobin and F_I_O_2_ had minimal effect on P_a_CO_2_. The effect of cardiac output on P_a_CO_2_ was variable, depending on the shunt fraction in the lung.

P_a_CO_2_ decreased as extracorporeal circuit blood flow was increased, but the changes were relatively small in the range used clinically for vv-ECMO of > 2 l/min. P_a_CO_2_ decreased as gas flow to the oxygenator rose and increased with recirculation. The oxygen fraction of gas flow to the oxygenator had minimal effect on P_a_CO_2_.

**Conclusions:**

This mathematical model of gas exchange during vv-ECMO found that the main determinants of P_a_CO_2_ during vv-ECMO were pulmonary shunt fraction, metabolic CO_2_ production, gas flow to the oxygenator and extracorporeal circuit recirculation.

**Electronic supplementary material:**

The online version of this article (10.1186/s40635-018-0183-4) contains supplementary material, which is available to authorized users.

## Background

The use of extracorporeal respiratory support to manage severe respiratory failure is increasing [1]. When oxygenation is adequate and the main problem is hypercapnia, extracorporeal CO_2_ removal may be used, but if oxygenation is inadequate then venovenous extracorporeal membrane oxygenation (vv-ECMO) is required. Management of these patients requires a thorough understanding of vv-ECMO physiology, which is a complex physiological interaction between the cardio-respiratory system and the vv-ECMO circuit [2]. The main goal of vv-ECMO is to avoid hypoxia, but with consideration of minimising the important adverse effects associated with hyperoxia [3], hypercapnia and hypocapnia [4], all of which may occur during vv-ECMO. Understanding the factors that determine O_2_ exchange and CO_2_ exchange during vv-ECMO and safely manipulating the complex physiology is vital.

Clinical studies have examined O_2_ and CO_2_ exchange during vv-ECMO for respiratory failure [5]. Circuit blood flow was the main determinant of arterial oxygenation, while the sweep gas flow through the oxygenator was the main determinant of CO_2_ elimination. While clinical studies are important in exploring vv-ECMO physiology, mathematical modelling is an essential tool to extend understanding of physiological systems [6]. It enables study of dynamic scenarios that cannot be examined in clinical studies due to ethical considerations, the inability to isolate the effects of changing a single parameter due to reflex responses and the logistics of clinical studies in a limited number of patients.

Hollow fibre membrane oxygenators have become the standard of care for vv-ECMO [7]. Mathematical models of O_2_ and CO_2_ exchange in these devices have been developed [8, 9], and provide useful information about how they behave. However, these models only examine oxygenator function, and do not consider its interaction with the cardio-respiratory system, so their clinical utility is limited. The determinants of oxygenation during vv-ECMO have been explored with mathematical models that incorporate both an oxygenator and the cardiorespiratory physiology and their findings fit well with clinical studies [10, 11], but these models have not incorporated CO_2_ exchange.

The purpose of this work was to develop a new model of the cardiorespiratory system during vv-ECMO, which incorporated O_2_, CO_2_ and N_2_ exchange, in both the oxygenator and the lung. It was used to assess the effects of a various physiological parameters and vv-ECMO circuit settings on vv-ECMO physiology.

## Methods

### Model description

**Fig. 1:**
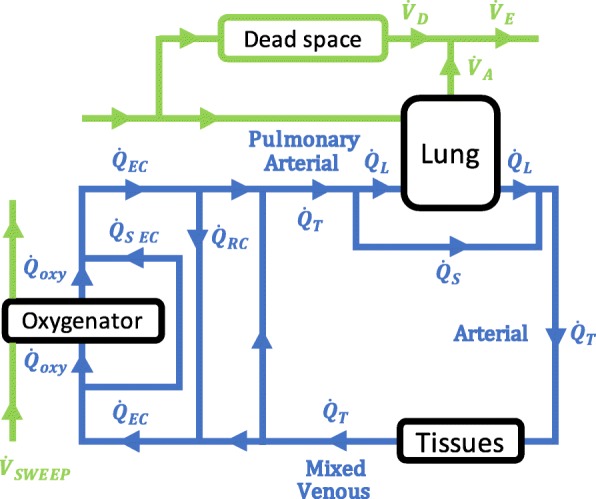
Outline of the ECMO model. Blood flow shown in blue. ${\dot{Q}}_T$ is cardiac output, ${\dot{Q}}_L$ is blood flow to lung compartment, ${\dot{Q}}_S$ is pulmonary shunt blood flow, ${\dot{Q}}_{EC}$ is blood flow to the extracorporeal blood circuit,${\dot{Q}}_{oxy}$ is blood flow to the ideal oxygenator compartment that participates in gas exchange, while ${\dot{Q}}_{S\  EC}$ is shunt blood flow that does not participate in gas exchange. ${\dot{Q}}_{EC}$ includes both recirculated blood flow (${\dot{Q}}_{RC}\Big)$ and part of the “mixed venous” blood flow from the tissues. Gas flows shown in green. ${\dot{V}}_A$ is alveolar ventilation, ${\dot{V}}_D$ is dead space ventilation, ${\dot{V}}_E$ is expired minute ventilation, ${\dot{V}}_{\mathrm{SWEEP}}$ is sweep gas flow to the oxygenator, oxygenator and lung are modelled as ideal lung compartments. Tissues consume O_2_ and produce CO_2_. “Arterial” blood perfuses the tissues, and “Mixed Venous” blood drains from the tissues. “Pulmonary Arterial” blood is a mixture of blood that has passed through the extracorporeal circuit, and blood that has not

A simple model of the cardiorespiratory system during vv-ECMO (Fig. 1) was implemented using Matlab R2017a (Mathworks, USA). A more detailed description of the model, including the mathematical equations underlying it, is given in the Additional file 1.

A three-compartment model of the lung, based on Riley and Cournard’s model [12], was used. One compartment was “shunt”, with perfusion but no ventilation, and did not participate in gas exchange. The second compartment was the “ideal lung” compartment, in which pulmonary gas exchange occurred. Diffusion equilibrium was assumed. The third compartment was “dead space”, with ventilation but no perfusion, and did not participate in gas exchange. Kelman’s subroutines [13–15] were used to calculate contents from partial pressures. The equations for gas exchange were based on those used by West and Wagner [16]. The model examines the system at equilibrium. Because of the presence of the oxygenator, N_2_ exchange across the lung was not assumed to be zero. Expired minute ventilation (${\dot{V}}_E$) and expired alveolar ventilation (${\dot{V}}_A$) were expressed at body temperature and pressure saturated (BTPS), and contents of gases dissolved in blood were expressed as standard temperature and pressure dry (STPD).

The oxygenator was modelled in a similar way to the lung, except that the shunt fraction and the dead space were set to zero, and recirculation was incorporated. Diffusion equilibrium was assumed in the “ideal oxygenator” compartment. Sweep gas flow to the oxygenator (${\dot{V}}_{\mathrm{SWEEP}}$) was expressed at ambient temperature (24 °C) and pressure (760 mmHg) dry (ATPD).

Input parameters required by the model are total cardiac output in l/min (${\dot{Q}}_T$), pulmonary shunt fraction $\left(\frac{{\dot{Q}}_S}{{\dot{Q}}_T}\right)$, inspired oxygen fraction to the lung (F_I_O_2_), blood flow to the extracorporeal circuit in l/min (${\dot{Q}}_{EC}\Big)$, fraction of ${\dot{Q}}_{EC}$ that is recirculated to the extracorporeal circuit $\left(\frac{{\dot{Q}}_{RC}}{{\dot{Q}}_{EC}}\right)$, shunt fraction of the extracorporeal circuit $\left(\frac{{\dot{Q}}_{s\  EC}}{{\dot{Q}}_{EC}}\right)$, oxygen fraction of gas flowing to the oxygenator (*F*_*OXY*_O_2_), sweep gas flow to the oxygenator in l/min ATPD (${\dot{V}}_{\mathrm{SWEEP}}$), haemoglobin in g/dl (*Hb*), body temperature in degrees Celsius (temp), a factor that accounts for shifts in the O_2_ dissociation curve due to changes in 2,3 di-phosphoglycerate (DP50), oxygen consumption in ml STPD/min ($\dot{V}{\mathrm{O}}_2\Big)$ and respiratory quotient (RQ). The base excess was assumed to be zero. Haematocrit (Hct) was calculated as 3 × Hb / 100.

The program uses initial trial values of P_pa_O_2_, P_pa_CO_2_ and P_pa_N_2_ (gas partial pressures in pulmonary arterial blood). The principle of conservation of mass for O_2_, CO_2_ and N_2_ allows the partial pressures and contents in each of the domains of the model to be calculated sequentially, including calculated estimates of P_pa_O_2_, P_pa_CO_2_ and P_pa_N_2_, that follow from the trial values. The mathematical problem is considered solved when trial values of P_pa_O_2_, P_pa_CO_2_ and P_pa_N_2_ have been found that generate calculated estimates that are all within 0.001 mmHg of the trial values. The problem is essentially that of finding the root in three dimensions of a system of non-linear equations which is solved using the false position method, in three dimensions.

To examine the physiological interactions between vv-ECMO settings and the cardiorespiratory system during vv-ECMO, the lung in the model was set up to mimic a patient with varying severity of acute respiratory distress syndrome (ARDS). The approach taken was based on Gattonini’s concept of the ARDS lung consisting of a “baby lung” with near normal specific compliance and dependent areas of atelectasis the size of which corresponds to the shunt fraction [17]. Contemporary approaches to mechanical ventilation limit the distending pressure the lung is exposed to, so ${\dot{V}}_A$ falls, as the amount of atelectasis and corresponding pulmonary shunt fraction increases. This was modelled by keeping the ventilation perfusion ratio of the baby lung $\left(\frac{{\dot{V}}_A}{{\dot{Q}}_L}\right)$ at a constant value, mimicking the clinical situation where ${\dot{V}}_A$ falls and $\frac{V_D}{V_T}$ rises with worsening ARDS. To achieve this, ${\dot{V}}_D$ was maintained at a constant value, and ${\dot{V}}_E$ varied to achieve the desired value of $\frac{{\dot{V}}_A}{{\dot{Q}}_L}$.

The ${\dot{V}}_E$ required to give a P_a_CO_2_ of 40 mmHg, when the lung had no shunt and $\frac{V_D}{V_T}$ was 0.3, was initially calculated. In the standard ARDS lung, this value of ${\dot{V}}_E$ was 6.157 l/min, ${\dot{V}}_D$ was 1.847 l/min and the corresponding ventilation-perfusion ratio of perfused lung $\left(\frac{{\dot{V}}_A}{{\dot{Q}}_L}\right)$ was 0.7183. In keeping with the baby lung concept, $\frac{{\dot{V}}_A}{{\dot{Q}}_L}$ was maintained constant at 0.7183, while $\frac{{\dot{Q}}_S}{{\dot{Q}}_T}$ was varied between 0.5 and 1. ${\dot{V}}_D$ was maintained constant at 1.847 l/min allowing the $\frac{V_D}{V_T}$ and ${\dot{V}}_E$ for any given scenario to be calculated. Total atelectasis of the lung is common during lung protective ventilation on vv-ECMO for severe ARDS, and this is represented in the model when $\frac{{\dot{Q}}_S}{{\dot{Q}}_T}$ is 1.0.

Where the model would require ${C}_{\overline{v}}{\mathrm{O}}_2$ < 0 for a solution, this was considered physiological untenable and the program returned an error warning and no results were output. Results are presented with the upper and lower values on the *x* axis scale set to encompass the *x* axis values tested, so missing data are due to an untenable result.

### Scenarios examined with the model

First, a validation was performed by comparing the findings of the model about oxygenation to those of Zanella’s model [11]. Input parameters were set to those in Zanella’s paper, and ${\dot{V}}_{\mathrm{SWEEP}}$ adjusted to obtain P_a_CO_2_ of 40 mmHg. The same output parameters were examined, enabling validation by reproducing the graphs from Zanella’s paper.

For all subsequent modelling, unless otherwise stated, F_I_O_2_ 1.0, *F*_*OXY*_O_2_ 1.0, ${\dot{Q}}_T$ 6 l/min, $\dot{V}{\mathrm{O}}_2$ 250 ml/min STPD, Hb 10 g/dl, DP50 0, $\frac{{\dot{Q}}_{s\  EC}}{{\dot{Q}}_{EC}}$ 0, $\frac{{\dot{Q}}_{RC}}{{\dot{Q}}_{EC}}$ 0, temp 37 °C, *RQ* 0.8 and${\dot{V}}_D$ 1.847 l/min were used as input parameters. ${\dot{Q}}_{EC}$, $\frac{{\dot{Q}}_S}{{\dot{Q}}_T}$ and ${\dot{V}}_{\mathrm{SWEEP}}$ were set and varied as required for the scenario being simulated. The derived parameters $\frac{{\dot{V}}_A}{{\dot{Q}}_L}$ and ${\dot{V}}_D$ were maintained constant at 0.7183 and 1.847 l/min. The output of the program for each scenario was written to a .csv file (these have been combined into the Additional file 2).

Second, the effect on oxygenation of different approaches to managing ${\dot{V}}_{\mathrm{SWEEP}}$ was investigated. The standard ARDS lung was used with $\frac{{\dot{Q}}_S}{{\dot{Q}}_T}$ ranging from 0.5 to 1.0. Three ways in which ${\dot{V}}_{\mathrm{SWEEP}}$ could be managed as ${\dot{Q}}_{EC}$ was varied were examined: (a) ${\dot{V}}_{\mathrm{SWEEP}}$ adjusted to maintain arterial P_a_CO_2_ = 40 mmHg; (b) ${\dot{V}}_{\mathrm{SWEEP}}$ fixed at 2.5, 5, 10 and 15 l/min; and (c) ${\dot{V}}_{\mathrm{SWEEP}}$ adjusted to maintain $\frac{{\dot{V}}_{\mathrm{SWEEP}}\ }{{\dot{Q}}_{EC}}$ constant.

Third, the factors that affect P_a_CO_2_ in the patient on vv-ECMO were studied using the standard ARDS lung.

Finally, the sensitivity of the output from the model to the choice of parameters set in the model lung was examined. The standard ARDS lung model was chosen to mimic a lung where the shunt fraction parallels the development of atelectactic lung, which is perfused but unventilated. ${\dot{V}}_A$ is proportional to the amount of non-atelectatic lung.

$$$$ {\dot{V}}_A={{\dot{V}}_A}{~}_{\left[{\dot{Q}}_s/{\dot{Q}}_T=0\right]}\times \left(1-\frac{{\dot{Q}}_S}{{\dot{Q}}_T}\right),\mathrm{so}\frac{{\dot{V}}_A}{{\dot{Q}}_L}\mathrm{is}\ \mathrm{constant}. $$

The other extreme is a lung in which ${\dot{V}}_A$ remains constant regardless of $\frac{{\dot{Q}}_S}{{\dot{Q}}_T}$ (constant ${\dot{V}}_A$ model). $\frac{{\dot{V}}_A}{{\dot{Q}}_L}=\frac{{\dot{V}}_A}{{\dot{Q}}_T\left(1-\frac{{\dot{Q}}_S}{{\dot{Q}}_T}\right)}$. The constant ${\dot{V}}_A$ model was modelled with ${\dot{V}}_A$ maintained constant at 4.310 l/min. This was the ${\dot{V}}_A$ that gave P_a_CO_2_ of 40 mmHg when the lung had no shunt. The effect on P_a_CO_2_ of changing ${\dot{Q}}_{EC}$, over a range of $\frac{{\dot{Q}}_S}{{\dot{Q}}_T}$, was examined with both the standard ARDS lung and the constant ${\dot{V}}_A$ model, and the results of the two models compared.

## Results

First, when the validation was performed with input parameters similar to Zanella’s model [11], the model produced almost identical graphical output (see the Additional file 3). This provided independent verification of Zanella’s model, and validation of the more complex mathematical approach that we have taken.

**Fig. 2:**
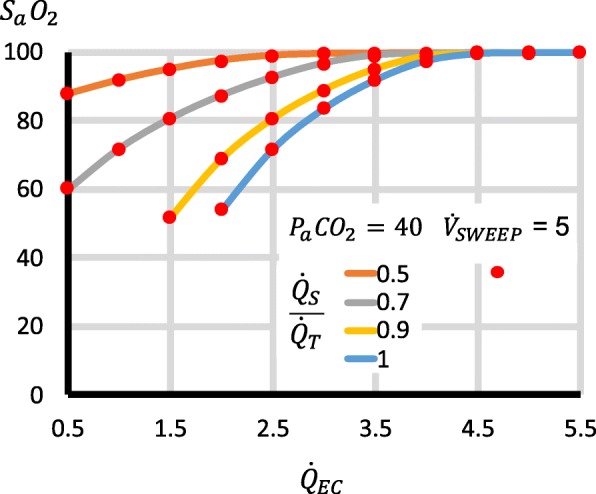
Effect on S_a_O_2_ of different approaches to managing ${\dot{V}}_{\mathrm{SWEEP}}$. Results for when P_a_CO_2_ is held constant at 40 mmHg by varying ${\dot{V}}_{\mathrm{SWEEP}}$ (continuous lines) compared to when ${\dot{V}}_{\mathrm{SWEEP}}$is held constant at 5 l/min (data points as red markers). As ${\dot{Q}}_{EC}$ increases, S_a_O_2_ rises. The higher the $\frac{{\dot{Q}}_S}{{\dot{Q}}_T}$, the higher the ${\dot{Q}}_{EC}$ required to achieve “acceptable” values of *S*_*a*_*O*_2_ > 85%. The red dots of fixed ${\dot{V}}_{\mathrm{SWEEP}}$ = 5 l/min match the curves of variable ${\dot{V}}_{\mathrm{SWEEP}}$ (fixed P_a_CO_2_), demonstrating that the approach taken to managing ${\dot{V}}_{\mathrm{SWEEP}}$ has minimal effect on S_a_O_2_

Second, when the effect on oxygenation of different approaches to managing ${\dot{V}}_{\mathrm{SWEEP}}$ was investigated, the way in which ${\dot{V}}_{\mathrm{SWEEP}}$ was managed as ${\dot{Q}}_{EC}$ varied had minimal effect on oxygenation (Fig. 2 and Additional file 4: Figure S1). As ${\dot{Q}}_{EC}$ increased, S_a_O_2_ and ${\mathrm{S}}_{\overline{\mathrm{v}}}{\mathrm{O}}_2$ rose. The higher the $\frac{{\dot{Q}}_S}{{\dot{Q}}_T}$, the higher the ${\dot{Q}}_{EC}$ to achieve “acceptable” values of S_a_O_2_ >  85% and ${\mathrm{S}}_{\overline{\mathrm{v}}}{\mathrm{O}}_2$ > 60%. For all these scenarios, at any given ${\dot{Q}}_{EC}$ and $\frac{{\dot{Q}}_S}{{\dot{Q}}_T}$, the output S_a_O_2_ and ${\mathrm{S}}_{\overline{\mathrm{v}}}{\mathrm{O}}_2$ did not differ by more than 1.5 (% oxygen saturation) from the S_a_O_2_ and ${\mathrm{S}}_{\overline{\mathrm{v}}}{\mathrm{O}}_2$ output in the simulation that held P_a_CO_2_ constant at 40 mmHg. Output C_a_O_2_ did not differ by more than 0.2 (ml STPD/100 ml blood). The biggest difference in P_a_O_2_ was 19 mmHg, which occurred at high ${\dot{Q}}_{EC}$ when P_a_O_2_ was > 500 mmHg.

**Fig. 3:**
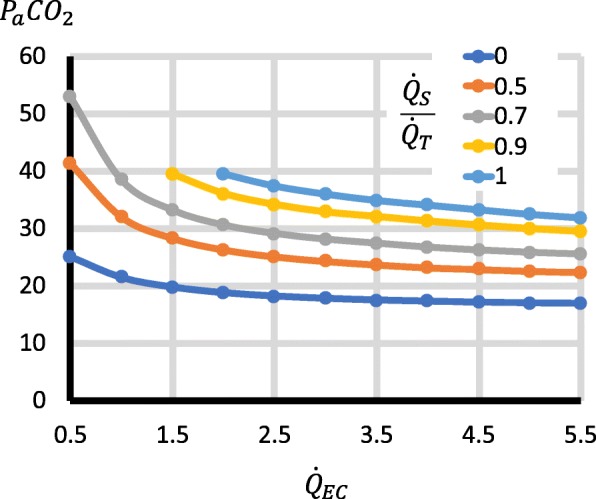
Effect on P_a_CO_2_ of ${\dot{\mathrm{Q}}}_{\mathrm{EC}}$ and $\frac{{\dot{Q}}_S}{{\dot{Q}}_T}$. ${\dot{V}}_{\mathrm{SWEEP}}$ is held constant at 5 l/min. As ${\dot{Q}}_{EC}$ is reduced, P_a_CO_2_ rises, but at > 2 l/min the curve is relatively flat, particularly when $\frac{{\dot{Q}}_S}{{\dot{Q}}_T}$ is low. P_a_CO_2_ rises as $\frac{{\dot{Q}}_S}{{\dot{Q}}_T}$ increases

**Fig. 4:**
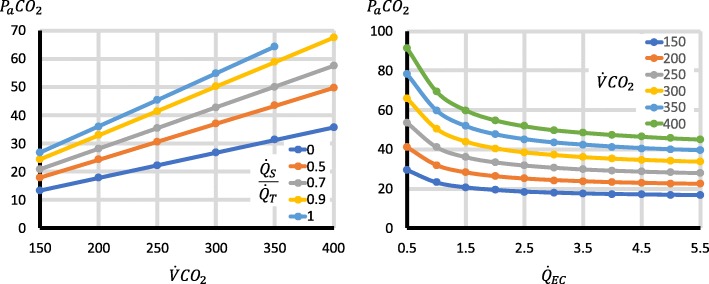
Effect on P_a_CO_2_ of $\dot{V}{CO}_2$. Left graph is for a range of $\frac{{\dot{Q}}_S}{{\dot{Q}}_T}$, with ${\dot{Q}}_{EC}$ = 3 l/min and ${\dot{V}}_{\mathrm{SWEEP}}$ = 5 l/min. There is a linear relationship between $\dot{V}{\mathrm{CO}}_2$ and P_a_CO_2_, which is steeper at high pulmonary shunt fractions. Right graph is for a range of ${\dot{Q}}_{EC}$, with $\frac{{\dot{Q}}_S}{{\dot{Q}}_T}=0.5$ and ${\dot{V}}_{\mathrm{SWEEP}}$ = 5 l/min. P_a_CO_2_ rises as ${\dot{Q}}_{EC}$ falls, but for ${\dot{Q}}_{EC}$ above 2 l/min, the changes are relatively small, but become larger with higher $\dot{V}{\mathrm{CO}}_2$

**Fig. 5:**
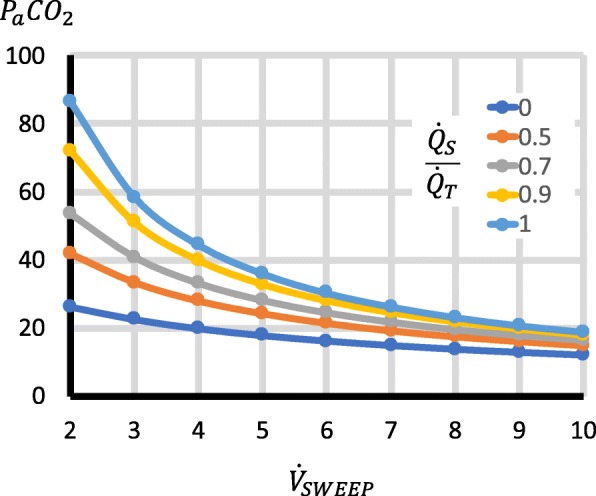
Effect on P_a_CO_2_ of ${\dot{V}}_{\mathrm{SWEEP}}$ and $\frac{{\dot{Q}}_S}{{\dot{Q}}_T}$. ${\dot{Q}}_{EC}$ is held constant at 3 l/min. As ${\dot{V}}_{\mathrm{SWEEP}}$ is reduced, P_a_CO_2_ rises, with a steeper rate of rise when $\frac{{\dot{Q}}_S}{{\dot{Q}}_T}$ is high. P_a_CO_2_ rises as $\frac{{\dot{Q}}_S}{{\dot{Q}}_T}$increases

**Fig. 6:**
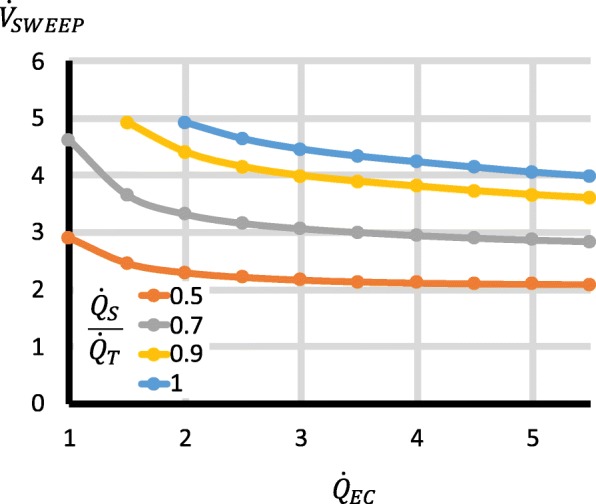
Effect on $the\ {\dot{V}}_{\mathrm{SWEEP}}$ required to maintain P_a_CO_2_ at 40 mmHg, of ${\dot{Q}}_{EC}$ and $\frac{{\dot{Q}}_S}{{\dot{Q}}_T}$. As ${\dot{Q}}_{EC}$ is reduced, the required ${\dot{V}}_{\mathrm{SWEEP}}$ rises, though the rise is small until ${\dot{Q}}_{EC}$ is < 2 l/min. The required ${\dot{V}}_{\mathrm{SWEEP}}$ rises as $\frac{{\dot{Q}}_S}{{\dot{Q}}_T}$ rises

**Fig. 7:**
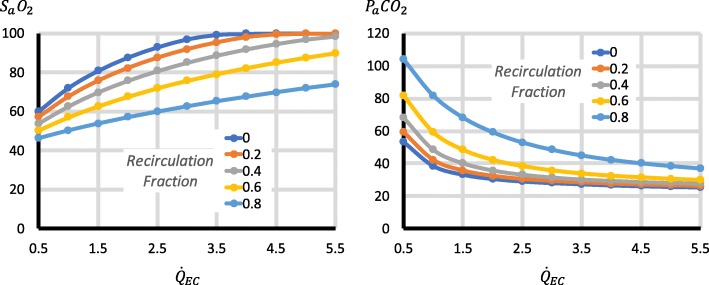
The effect of recirculation. $\frac{{\dot{Q}}_S}{{\dot{Q}}_T}$ is held constant at 0.7. With increasing recirculation, P_a_CO_2_ rises and S_a_O_2_ falls. Recirculation has more effect on *P*_*a*_*CO*_2_ when ${\dot{Q}}_{EC}$ is low

Third, the factors with important effects on P_a_CO_2_ were $\frac{{\dot{Q}}_S}{{\dot{Q}}_T}$, ${\dot{Q}}_{EC}$, ${\dot{V}}_{\mathrm{SWEEP}}$, $\dot{V}{\mathrm{CO}}_2$ and $\frac{{\dot{Q}}_{RC}}{{\dot{Q}}_{EC}}$. Higher $\frac{{\dot{Q}}_S}{{\dot{Q}}_T}$ increased P_a_CO_2_ (Fig. 3). Increases in ${\dot{Q}}_{EC}$ reduced P_a_CO_2_, but for ${\dot{Q}}_{EC}$ > 2 l/min, the changes were relatively small (Fig. 3), though they became larger with high $\dot{V}{\mathrm{CO}}_2$ (Fig. 4). Increases in ${\dot{V}}_{\mathrm{SWEEP}}$ reduced P_a_CO_2_ (Fig. 5). The ${\dot{V}}_{\mathrm{SWEEP}}$ required to maintain P_a_CO_2_ at 40 mmHg fell as ${\dot{Q}}_{EC}$ increased, but for ${\dot{Q}}_{EC}$ > 2 l/min, the changes were relatively small (Fig. 6). There was a linear increase in P_a_CO_2_ with $\dot{V}{\mathrm{CO}}_2$ (Fig. 4). P_a_CO_2_ rose and S_a_O_2_ fell as recirculation increased (Fig. 7). At high $\frac{{\dot{Q}}_S}{{\dot{Q}}_T}$, when the recirculation was high, S_a_O_2_ fell to physiological untenable levels (Additional file 5: Figure S2).

Changes in Hb, F_I_O_2_ and F_OXY_O_2_, had minimal effect on P_a_CO_2_, despite the important effects these parameters have on oxygenation (Additional file 6: Figure S3, Additional file 7: Figure S4, and Additional file 8: Figure S5). The effect of ${\dot{Q}}_T$ on P_a_CO_2_ was variable, depending on the shunt fraction (Additional file 9: Figure S6).

Finally, the sensitivity of the model to the parameters set in the model lung was studied. With both the constant ${\dot{V}}_A$ lung model and the standard ARDS lung model, increases in ${\dot{Q}}_{EC}$ produced a fall in P_a_CO_2_, but for ${\dot{Q}}_{EC}$ > 2 l/min, the changes were relatively small. The results for $\frac{{\dot{Q}}_S}{{\dot{Q}}_T}$ of 0, and for $\frac{{\dot{Q}}_S}{{\dot{Q}}_T}$ of 1 were identical, but the curves for $\frac{{\dot{Q}}_S}{{\dot{Q}}_T}$ between these values were in different positions (Additional file 10: Figure S7).

## Discussion

Previous mathematical models of gas exchange during vv-ECMO have only examined O_2_ exchange [10, 11], or made unrealistic assumptions about CO_2_ exchange [18]. They are unable to assess the determinants of CO_2_ exchange. To overcome this limitation, a mathematical model of gas exchange during vv-ECMO, that incorporated O_2_, CO_2_ and N_2_ exchange, was developed. The effect of input physiological parameters and vv-ECMO circuit settings on oxygenation was compared to the findings of Zanella’s model [11], which only included O_2_ exchange. Our model closely reproduced the outputs of Zanella’s model and can be reviewed in the Additional files. This provides validation of the more complex mathematical approach presented here. This validation is limited to the effects on oxygenation, as Zanella did not examine CO_2_ exchange. Zanella’s simpler approach would be preferred if only O_2_ exchange was of interest, but it cannot be used if CO_2_ exchange is to be examined.

The major advantage of this model over previous models is the incorporation of CO_2_ exchange, which allows the effect of input physiological parameters and vv-ECMO circuit settings on P_a_CO_2_ to be assessed. The key finding of our paper is that providing ${\dot{Q}}_{EC}$ ≥ 2 l/min, the major determinants of P_a_CO_2_ are $\dot{V}{\mathrm{CO}}_2$, ${\dot{V}}_{\mathrm{SWEEP}}$ and $\frac{{\dot{Q}}_S}{{\dot{Q}}_T}.$ This provides guidance to the clinician who wishes to control P_a_CO_2_. ${\dot{V}}_{\mathrm{SWEEP}}$ can be adjusted by changing the sweep gas flow rate on the oxygenator, and $\dot{V}{\mathrm{CO}}_2$ can be modified with sedation, paralysis and temperature control. $\frac{{\dot{Q}}_S}{{\dot{Q}}_T}$ is more dependent on the underlying lung pathology. Manipulation of Hb, F_I_O_2_, *F*_*OXY*_O_2_ and ${\dot{Q}}_T$ will have minimal effect on P_a_CO_2_.

While ultra-protective ventilation strategies have been recommended for patients on vv-ECMO [19], practices vary widely between centres [20]. The effect of different ventilation strategies was not studied with the model. Unless $\frac{{\dot{Q}}_S}{{\dot{Q}}_T}$ is 1, differences in minute ventilation that modify ${\dot{V}}_A$ will directly affect P_a_CO_2_. Differences in positive end expiratory pressure may affect lung recruitment and alter $\frac{{\dot{Q}}_S}{{\dot{Q}}_T}$.

Schmidt et al. observed in vivo the determinants of oxygenation and decarboxylation during vv-ECMO in ten adult patients with respiratory failure [5]. When ${\dot{Q}}_{EC}$ was reduced from a baseline of 5.8 ± 0.8 l/min by 40% to 2.4 ± 0.3 l/min, P_a_O_2_ and S_a_O_2_ fell, but there was no change in P_a_CO_2_. When maintaining ${\dot{Q}}_{EC}$ at baseline values, reducing *F*_*OXY*_O_2_ from 1.0 to 0.4 resulted in a fall in P_a_O_2_ and S_a_O_2_, but no change in P_a_CO_2_. In contrast, when the sweep gas flow rate reduced from 10 to 2 l/min, P_a_O_2_ did not change, and P_a_CO_2_ rose. In the three patients given a red blood cell transfusion, there was a rise in P_a_O_2_ (no data were presented for P_a_CO_2_). These findings are consistent with the predictions of the physiology made by the mathematical model, providing clinical validation of the model.

The model has several limitations. Gas exchange in the oxygenator was modelled as perfusion limited, which is not always the case with a real oxygenator. When the surface area of a membrane lung is low and blood flow is high, CO_2_ elimination is limited by diffusion. As the surface area increases, diffusion limitation becomes less important and CO_2_ elimination becomes predominantly perfusion limited [21]. O_2_ uptake increases linearly with blood flow in the Quadrox oxygenator, up to the rated maximum ${\dot{Q}}_{EC}$ of 7 l/min [9], though this is not the case for all oxygenators. The assumption of predominantly perfusion limited gas exchange is unlikely to result in major inaccuracy during vv-ECMO, providing a suitable oxygenator is used. Oxygenator performance deteriorates with time, due to deposition of protein and clot formation on the surface exposed to blood, and water accumulation on the surface exposed to gas [22]. With long-term use, the findings of the model may become less applicable as the oxygenator performance deteriorates. Input parameters of the model were treated as independent variables, which is not true in vivo. For example, cardiac output changes in response to anaemia, hypercapnia or hypoxia. $\frac{{\dot{Q}}_S}{{\dot{Q}}_T}$ changes in response to P_pa_O_2_ which affects pulmonary vascular tone. Despite these limitations, the findings of the model are in keeping with in vivo data [5].

The model does not include a dead space compartment in the oxygenator. Castagna et al. studied oxygenators after several days of vv-ECMO (4.0 days, IQR 2.0–8) and found $\frac{{\dot{V}}_D}{{\dot{V}}_T}$ of 47.8 ± 15.3 (mean ± sd) [22]. This finding fits the clinical observation that the sweep gas flow rate required to maintain normocapnia often gradually increases during an ECMO run. As dead space develops, the same gas transfer across the oxygenator can be maintained by increasing ${\dot{V}}_{\mathrm{SWEEP}}$.

The findings presented in this paper relate to the ARDS patient with high $\frac{{\dot{Q}}_S}{{\dot{Q}}_T}$, that requires ${\dot{Q}}_{EC}$ ≥ 2 l/min to support oxygenation. During extracorporeal CO_2_ removal, ${\dot{Q}}_{EC}$ is usually < 2 l/min, and ${\dot{Q}}_{EC}$ becomes a major determinant of CO_2_ elimination. Gas exchange across the membranes used for extracorporeal CO_2_ removal is often limited by incomplete diffusion [21], and this may be exacerbated by the high gas sweep to blood flow ratios that are used to facilitate CO_2_ removal. A range of techniques have been tried to enhance CO_2_ removal including using carbonic anhydrase [23], acidification of blood or dialysate [24] and electrodialysis [25]. Further modelling of the factors affecting gas exchange during extracorporeal CO_2_ removal may prove enlightening.

Consider a scenario where a patient that was stable on ECMO becomes febrile and hypoxic. As the metabolic rate increased with the fever, the sweep gas was increased to maintain normal P_a_CO_2_. To correct the hypoxia, it is planned to increase ${\dot{Q}}_{EC}$ from 2.5 to 5 l/min. What should be done with ${\dot{V}}_{\mathrm{SWEEP}}$ to maintain normal P_a_CO_2_? The model predicts that with normal $\dot{V}{\mathrm{CO}}_2$, changes in ${\dot{Q}}_{EC}$ would have minimal effect on P_a_CO_2_ providing ${\dot{Q}}_{EC}$ is > 2 l/m, so no change in ${\dot{V}}_{\mathrm{SWEEP}}$ would be required. This is consistent with standard teaching on managing ECMO. However, in this scenario, $\dot{V}{\mathrm{CO}}_2$ is elevated, and the model predicts that increasing ${\dot{Q}}_{EC}$ will increase CO_2_ elimination, so the clinician will need to reduce ${\dot{V}}_{\mathrm{SWEEP}}$ to maintain a normal P_a_CO_2_. Another scenario where the model may be of assistance to the clinician is when a patient is anaemic on ECMO and requires blood transfusion to maintain adequate oxygenation. What should be done with ${\dot{V}}_{\mathrm{SWEEP}}$ to maintain normal P_a_CO_2_? Despite the importance of Hb in CO_2_ transport in blood, the model predicts that during vv-ECMO, even large changes in Hb will have minimal effect on P_a_CO_2_. No change in ${\dot{V}}_{\mathrm{SWEEP}}$ is required. The emphasis of this paper has been on CO_2_ exchange during vv-ECMO, but the model is equally applicable to O_2_ exchange. It supports clinical decision making by improving the clinician’s understanding of vv-ECMO physiology.

## Conclusion

A mathematical model of gas exchange during vv-ECMO, incorporating O_2_, CO_2_ and N_2_ exchange, was developed. The results of the model were consistent with previous mathematical models that only examined O_2_ exchange and predict the findings of clinical studies. The main determinants of P_a_CO_2_ during vv-ECMO were lung $\frac{{\dot{Q}}_S}{{\dot{Q}}_T}$, metabolic CO_2_ production, ${\dot{V}}_{\mathrm{SWEEP}}$ and extracorporeal circuit recirculation.

## Additional files

Additional file 1: Description of the ECMO model and the mathematical equations on which it is based. (PDF 205 kb)

Additional file 2: This file contains the output of the program for the scenarios modelled. Each worksheet contains the output for a single scenario. (XLSX 236 kb)

Additional file 3: This file contains the output of the program for the validation against Zanella’s model. Each worksheet contains the output of the program and graphs generated from this output, corresponding to one of Zanella’s figures. (XLSX 155 kb)

Additional file 4:**Figure S1.** Shows the “Effect on ${S}_{\overline{v}}{O}_2$ of different approaches to managing ${\dot{V}}_{SWEEP}$”. (PDF 77 kb)

Additional file 5:**Figure S2.** Shows the “Effect of recirculation at high $\frac{{\dot{Q}}_S}{{\dot{Q}}_T}$*”. (PDF 67 kb)*

Additional file 6: **Figure S3.** Shows the “Effect on P_a_CO_2_ of Hb”. (PDF 74 kb)

Additional file 7: **Figure S4.** Shows the “Effect of F_I_O_2_ on P_a_CO_2_”. (PDF 74 kb)

Additional file 8: **Figure S5.** Shows the “Effect of *F*_*OXY*_*O*_2_ on P_a_CO_2_”. (PDF 76 kb)

Additional file 9:**Figure S6.** Shows the “Effect of changes in ${\dot{Q}}_T$ on P_a_CO_2_, over a range of $\frac{{\dot{Q}}_S}{{\dot{Q}}_T}$*”.* (PDF 72 kb)

Additional file 10: **Figure S7.** Shows the “Sensitivity to which lung model is used”. (PDF 88 kb)

## Abbreviations

$\dot{V}{\mathrm{O}}_2$: O_2_ consumption in ml STPD/min
$\dot{V}{\mathrm{CO}}_2$: CO_2_ production in ml STPD/min
C_a_O_2_: Arterial oxygen content in ml/dl blood STPD
S_a_O_2_: Arterial oxygen saturation
P_a_CO_2_: Arterial partial pressure of CO_2_ in mm Hg
P_a_O_2_: Arterial partial pressure of O_2_ in mm Hg
${\dot{Q}}_{\mathrm{EC}}$: Blood flow to the extracorporeal circuit in l/min
${\dot{Q}}_{\mathrm{T}}$: Cardiac output in l/min
${\dot{Q}}_{\mathrm{P}}$: Blood flow to the lung compartment in l/min
${\dot{Q}}_{\mathrm{S}}$: Pulmonary shunt blood flow in l/min
$\frac{V_D}{V_T}$: Dead space to tidal volume ratio
${\dot{V}}_D$: Dead space ventilation of the lung in l/min BTPS
${\dot{V}}_A$: Expired alveolar ventilation of the lung in l/min BTPS
${\dot{V}}_E$: Expired minute ventilation of the lung in l/min BTPS
$\frac{{\dot{V}}_A}{{\dot{Q}}_L}$: Expired ventilation perfusion ratio of the lung
$\frac{{\dot{Q}}_{RC}}{{\dot{Q}}_{EC}}$: Fraction of ${\dot{Q}}_{EC}$ that is recirculated to the extracorporeal circuit
F_I_O_2_: Inspired oxygen fraction of the lung
*F*_*OXY*_O_2_: Inspired oxygen fraction of the oxygenator
_CvO2_: Mixed venous oxygen content in ml/dl blood STPD
${S}_{\overline{v}}{O}_2$: Mixed venous oxygen saturation
P_pa_CO_2_: Partial pressure of CO_2_ in the pulmonary artery
P_pa_N_2_: Partial pressure of N_2_ in the pulmonary artery
P_pa_O_2_: Partial pressure of O_2_ in the pulmonary artery
$\frac{{\dot{Q}}_S}{{\dot{Q}}_T}$: Pulmonary shunt fraction
$\frac{{\dot{V}}_D}{{\dot{V}}_T}$: Ratio of dead space ventilation to total ventilation
$\frac{{\dot{Q}}_{s\ EC}}{{\dot{Q}}_{EC}}$: Shunt fraction of the extracorporeal circuit
${\dot{V}}_{\mathrm{SWEEP}}$: Sweep gas flow rate to the oxygenator in l/min ATPD
ARDS: Acute respiratory distress syndrome
ATPD: Ambient temperature and pressure dry
BTPS: Body temperature and pressure saturated
DP50: Factor that accounts for shifts in the O_2_ dissociation curve due to changes in 2,3 di-phosphoglycerate
Hb: Haemoglobin in g/dl
Hct: Haematocrit
STPD: Standard temperature and pressure dry
temp: Body temperature in °C
vv-ECMO: Venovenous extracorporeal membrane oxygenation
