# Supplementary material for: A mathematical model of CO2, O2 and N2 exchange during venovenous extracorporeal membrane oxygenation
Source: Intensive Care Med Exp. 2018 Aug 9;6:25. doi: 10.1186/s40635-018-0183-4 (PMC6085277; doi:10.1186/s40635-018-0183-4)
Supplement: Supplementary file 1 — Description of the ECMO model and the mathematical equations on which it is based. (PDF 205 kb) [file 40635_2018_183_MOESM1_ESM.pdf]

Description of the ECMO model and the mathematical equations on which it is based.

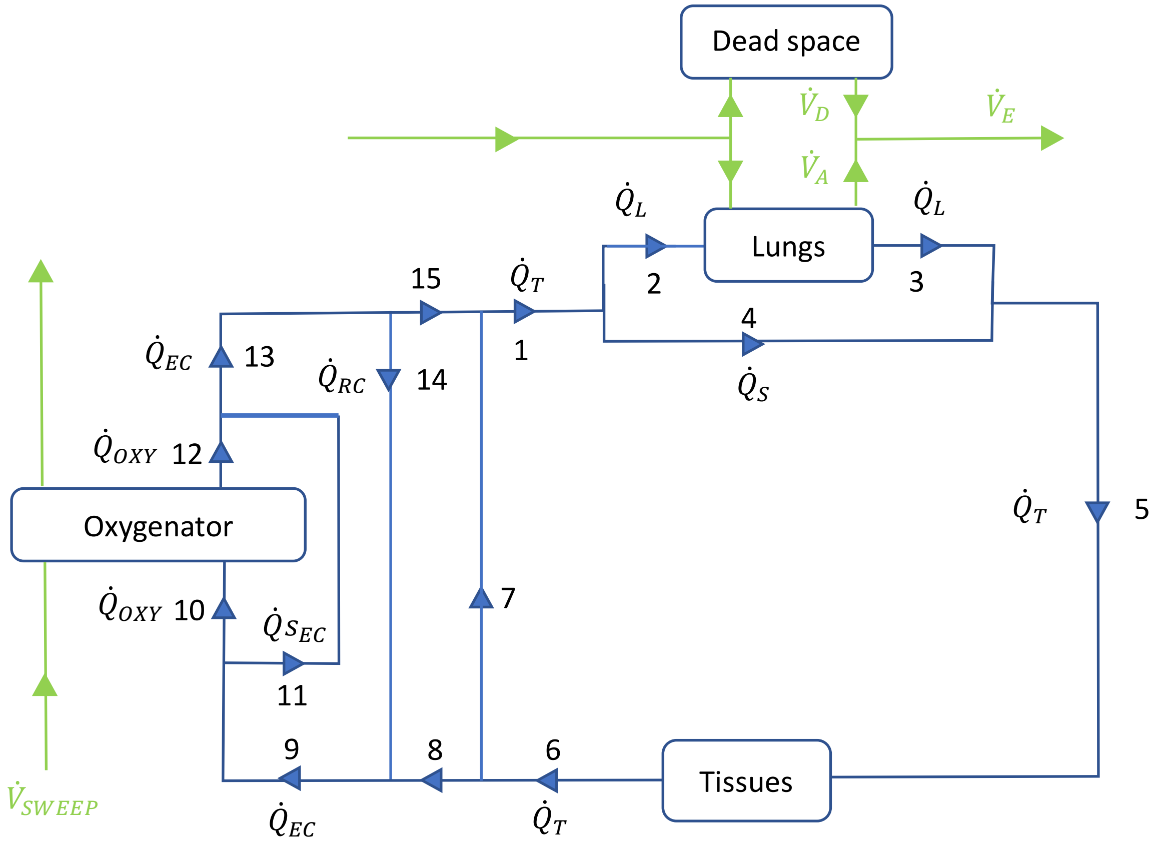

Figure 1. The ECMO model.
This is the model of veno-venous extracorporeal oxygenation used in the program. Blood flows are shown as blue. All the positions in the model with an associated blood flow are marked with a number and described below. Blood flow values in key positions are labelled for clarity.

$\dot{Q}_T$ is the cardiac output.
$\dot{Q}_L$ is the blood flow to the lung compartment.
$\dot{Q}_S$ is the blood flow to a shunt in parallel to the lung compartment.

$\dot{Q}_{EC}$ is the flow into and out of the extracorporeal circuit
$\dot{Q}_{OXY}$ is the blood flow to the oxygenator compartment.
$\dot{Q}s_{EC}$ is the blood flow to a shunt in parallel to the oxygenator compartment.
$\dot{Q}_{RC}$ is blood flow recirculated to the extracorporeal circuit.

Gas flows are shown as green

$\dot{V}_{SWEEP}$ is sweep gas flow to the oxygenator

$\dot{V}_A$ is alveolar ventilation
$\dot{V}_D$ is dead space ventilation
$\dot{V}_E$ is expired minute ventilation, and $\dot{V}_E = \dot{V}_A + \dot{V}_D$

Standard respiratory physiology practice is to express

- Blood gas contents at standard temperature and pressure dry per decilitre of blood
- Inspired, expired, and alveolar gas volumes as litre at body temperature and pressure saturated.

Sweep gas flow is given as ambient temperature (24° C) and pressure (760 mm Hg) dry.

Conversions are made between these units when required in the program.

1. The blood flow in the pulmonary artery is equal to the cardiac output $\dot{Q}_T$. This blood is a mixture of blood returning from the oxygenator, and blood returning from the body. The gas partial pressures in the pulmonary artery are $P_{pa}O_2$, $P_{pa}CO_2$, $P_{pa}N_2$, with contents of $C_{pa}O_2$, $C_{pa}CO_2$, $C_{pa}N_2$.

2. The blood flow to the lung is $\dot{Q}_L$ with the same gas partial pressures and contents as in the pulmonary artery.

3. The blood flow from the lung is $\dot{Q}_L$. The gases within it have fully equilibrated with the lung compartment, and the gas partial pressures are P_AO_2, P_ACO_2, P_AN_2, with contents of C_AO_2, C_ACO_2, C_AN_2. The equations for determining these gas partial pressures are described later.

4. The "shunt" blood flow in parallel to the blood flow to the lung is $\dot{Q}_S$ does not exchange blood with the lung compartment, and has the same gas partial pressures and contents as in the pulmonary artery.

5. The arterial blood flow is equal to the cardiac output $\dot{Q}_T$. The arterial gas partial pressures are P_aO_2, P_aCO_2, P_aN_2, with contents of C_aO_2, C_aCO_2, C_aN_2. Arterial blood is formed by the combination of blood flow through "normal" lung, and blood flow through the shunt, so that

$$\dot{Q}_T = \dot{Q}_L + \dot{Q}_S$$

$$C_aO_2 = \frac{C_AO_2 \,.\, \dot{Q}_L + C_{pa}O_2 \,.\, \dot{Q}_S}{\dot{Q}_T}$$

$$C_aCO_2 = \frac{C_ACO_2 \,.\, \dot{Q}_L + C_{pa}CO_2 \,.\, \dot{Q}_S}{\dot{Q}_T}$$

6. The "mixed venous" blood flow is equal to the cardiac output $\dot{Q}_T$. The tissues extract $\dot{V}O_2$ from the arterial blood and produce $\dot{V}CO_2$ which is added to the arterial blood.

$$C_{\bar{v}}O_2 = C_aO_2 - \frac{\dot{V}O_2}{\dot{Q}_T}$$

$$C_{\bar{v}}CO_2 = C_aCO_2 + \frac{\dot{V}CO_2}{\dot{Q}_T}$$

7. Part of the mixed venous blood flow passes to the pulmonary artery without exposure to the oxygenator. This blood flow is equal to $\dot{Q}_T - (\dot{Q}_{EC} - \dot{Q}_{RC})$.

8. The rest of the mixed venous blood flow passes to the oxygenator. The blood flow is equal to $\dot{Q}_{EC} - \dot{Q}_{RC}$.

9. The blood flow going into the extracorporeal circuit $\dot{Q}_{EC}$ is formed by the combination of mixed venous blood and post-oxygenator blood that has recirculated. The gas partial pressures in this blood are $P_{PREOXY}O_2$, $P_{PREOXY}CO_2$, $P_{PREOXY}N_2$, with contents of $C_{PREOXY}O_2$, $C_{PREOXY}CO_2$, $C_{PREOXY}N_2$.

$$C_{PREOXY}O_2 = C_{\bar{v}}O_2(\dot{Q}_{EC} - \dot{Q}_{RC}) + C_{POSTOXY}O_2.\dot{Q}_{RC}$$

$$C_{PREOXY}CO_2 = C_{\bar{v}}CO_2(\dot{Q}_{EC} - \dot{Q}_{RC}) + C_{POSTOXY}CO_2.\dot{Q}_{RC}$$

$$P_{PREOXY}N_2 = P_{\bar{v}}N_2(\dot{Q}_{EC} - \dot{Q}_{RC}) + P_{POSTOXY}N_2.\dot{Q}_{RC}$$

10. The effective blood flow to the oxygenator is $\dot{Q}_{OXY}$. This is analogous to $\dot{Q}_L$, and is the blood that will participate in gas exchange in the oxygenator. It has the same gas pressures and contents as $\dot{Q}_{EC}$
$\dot{Q}_{OXY} = \dot{Q}_{EC} - \dot{Q}s_{EC}$

11. The extracorporeal shunt flow $\dot{Q}s_{EC}$ is blood that passes to the oxygenator but does not participate in gas exchange. It has the same gas pressures and contents as $\dot{Q}_{EC}$.

12. The effective blood flow from the oxygenator is $\dot{Q}_{OXY}$. The gases within it have fully equilibrated with the membrane lung compartment, and the gas partial pressures are $P_{POSTOXY}O_2$, $P_{POSTOXY}CO_2$, $P_{POSTOXY}N_2$, with O_2 and CO_2 contents of $C_{POSTOXY}O_2$, $C_{POSTOXY}CO_2$. The equations for determining these gas partial pressures are described later and are essentially the same as for the lung.

13. The blood flow going out of the extracorporeal circuit $\dot{Q}_{EC}$ has O_2 and CO_2 contents of $C_{EC_{final}}O_2$, $C_{EC_{final}}CO_2$, and a partial pressure of N_2 of $P_{EC_{final}}N_2$. This blood is formed by the combination of blood that has participated in gas exchange with the oxygenator and blood that has not.

$$C_{EC_{final}}O_2 = \frac{C_{POSTOXY}O_2 \times \dot{Q}_{OXY} + C_{PREOXY}O_2 \times \dot{Q}s_{EC}}{\dot{Q}_{EC}}$$

$$C_{EC_{final}}CO_2 = \frac{C_{POSTOXY}CO_2 \times \dot{Q}_{OXY} + C_{PREOXY}CO_2 \times \dot{Q}s_{EC}}{\dot{Q}_{EC}}$$

$$P_{EC_{final}}N_2 = \frac{P_{POSTOXY}N_2 \times \dot{Q}_{OXY} + P_{PREOXY}N_2 \times \dot{Q}s_{EC}}{\dot{Q}_{EC}}$$

14. A proportion of the extracorporeal blood flow recirculates, and this blood flow $\dot{Q}_{RC}$ has the same contents and partial pressures as the blood leaving the extracorporeal circuit ($C_{EC_{final}}O_2$, $C_{EC_{final}}CO_2$, $P_{EC_{final}}N_2$).

15. The blood flow from the extracorporeal circuit that is not recirculated has contents and partial pressures of ($C_{EC_{final}}O_2$, $C_{EC_{final}}CO_2$, $P_{EC_{final}}N_2$).

Having described the rest of the model, we can now come back to the position marked as 1 on the diagram. The gas partial pressures in the pulmonary artery are $P_{pa}O_2$, $P_{pa}CO_2$, $P_{pa}N_2$, with contents of $C_{pa}O_2$, $C_{pa}CO_2$, $C_{pa}N_2$. The blood in the pulmonary artery is a mixture of blood returning from the oxygenator, and blood returning from the body, so that

$$C_{pa}O_2 = \frac{C_{EC_{final}}O_2\left(\dot{Q}_{EC} - \dot{Q}_{RC}\right) + C_{\bar{v}}O_2\,(\dot{Q}_T - (\dot{Q}_{EC} - \dot{Q}_{RC}))}{\dot{Q}_T}$$

$$C_{pa}CO_2 = \frac{C_{EC_{final}}CO_2\left(\dot{Q}_{EC} - \dot{Q}_{RC}\right) + C_{\bar{v}}CO_2\,(\dot{Q}_T - (\dot{Q}_{EC} - \dot{Q}_{RC}))}{\dot{Q}_T}$$

$$P_{pa}N_2 = \frac{P_{EC_{final}}N_2\left(\dot{Q}_{EC} - \dot{Q}_{RC}\right) + P_{\bar{v}}N_2\,(\dot{Q}_T - (\dot{Q}_{EC} - \dot{Q}_{RC}))}{\dot{Q}_T}$$

Mathematical methods used in the program

The lung and the oxygenator parts of the model contribute to calculation of pulmonary artery gas partial pressures, but also depend on them to make their calculations. This problem is overcome by using an iterative method. Initial trial values of $P_{pa}O_2$, $P_{pa}CO_2$ and $P_{pa}N_2$, are used. Calculations are made using these trial values, to make new estimates of $P_{pa}O_2$, $P_{pa}CO_2$ and $P_{pa}N_2$. The trial values and the new estimates are compared and accepted if within preset tolerances. If not, then they are used to generate new trial values, and the process continues to iterate. The false position method is the primary numerical method used for a root finding problem in three dimensions for a set of non-linear functions. The system is modelled at equilibrium.

Inputs to the program are

a) $\dot{Q}_T$ the cardiac output in l/min

b) Shunt Fraction of the lung $(\frac{\dot{Q}_S}{\dot{Q}_T})$, which is used with $\dot{Q}_T$ to calculate $\dot{Q}_L$ and $\dot{Q}_S$

   a. $\dot{Q}_S = \dot{Q}_T \cdot \frac{\dot{Q}_S}{\dot{Q}_T}$

   b. $\dot{Q}_L = \dot{Q}_T\,(1 - \frac{\dot{Q}_S}{\dot{Q}_T})$

c) F_IO_2 the inspired oxygen fraction of the lungs. It is assumed the inspired gas is O_2 and N_2.

d) $\dot{Q}_{EC}$ the blood flow to the extracorporeal circuit in l/min.

e) The fraction of $\dot{Q}_{EC}$ that is recirculated to the extracorporeal circuit (RCF_{EC})

f) The shunt fraction of the extracorporeal circuit $(\frac{\dot{Q}_{S\,EC}}{\dot{Q}_{EC}})$, which is used with $\dot{Q}_{EC}$ to calculate $\dot{Q}_{S_{EC}}$ and $\dot{Q}_{OXY}$.

   a. $\dot{Q}_{S_{EC}} = \dot{Q}_{EC} \cdot \frac{\dot{Q}_{S\,EC}}{\dot{Q}_{EC}}$

   b. $\dot{Q}_{OXY} = \dot{Q}_{EC}\left(1 - \frac{\dot{Q}_{S\,EC}}{\dot{Q}_{EC}}\right)$

g) $F_{OXY}O_2$ the oxygen fraction of the sweep gas flowing to the oxygenator

h) $\frac{\dot{V}}{\dot{Q}}oxy$ the expired ventilation perfusion ratio of the oxygenator.

i) Hb in g/dl. The haematocrit is expressed as a decimal fraction and set by

$$haematocrit = \frac{3 \times haemoglobin}{100}$$

j) Body temperature in degrees Celsius

k) DPG, which is a correction for shifts in the oxygen dissociation curve due to 2,3 di-phosphoglycerate.

l) $\dot{V}O_2$ the O_2 consumption of the body in ml STPD/dl of blood/min. $\dot{V}CO_2$ the CO_2 production of the body in ml STPD/dl blood/min, is set by

$$\dot{V}CO_2 = 0.8 \times \dot{V}O_2$$

Inputs j-m are required for Kelman's subroutines to calculate O_2 and O_2 contents from pO_2 and pCO_2, and vice versa. The effects of metabolic acidosis/alkalosis have not been incoporated, though this can be done with Kelman's subroutines.

It will be noted that there are significant similarities between this model, and West and Wagner's multi-compartment lung model that was used to examine the effects of ventilation perfusion mismatch, incorporating N_2 exchange. The major difference is that due to the presence of the oxygenator, West and Wagner's assumption that there is no N_2 exchange across the lung at equilibrium is not valid.

The program was initially run using iteration to find the expired minute ventilation ($\dot{V}_E$), dead space ventilation ($\dot{V}_D$), alveolar ventilation ($\dot{V}_A$) and lung ventilation perfusion ratio ($\frac{\dot{V}_A}{\dot{Q}_L}$) that satisfy the constraints of the model when P_aCO_2 is set at 40 mm Hg, $\frac{\dot{Q}_S}{\dot{Q}_T}$ is zero, $\frac{V_D}{V_T}$ is 0.3, and there is no extracorporeal blood flow. Other parameters used in this calculation were F_IO_2 1.0, $\dot{Q}_T$ 6 l/min, $\dot{V}O_2$ 250 ml/min STPD, *Hb* 10 g/dl, *DP50* 0, *temp* 37°C, and *RQ* 0.8. The values calculated were $\dot{V}_E$ of 6.157 L/min, $\dot{V}_D$ of 1.847 L/min, $\dot{V}_A$ of 4.310 L/min, and $\frac{\dot{V}_A}{\dot{Q}_L}$ of 0.7183. These calculated values of $\dot{V}_D$ and $\frac{\dot{V}_A}{\dot{Q}_L}$ were then maintained constant in all the scenarios modelled.

LUNG compartment

The lung is modelled as a single lung unit, with set expired minute ventilation $\dot{V}_A$, set perfusion $\dot{Q}_L$, and set F_IO_2. It is perfused by pulmonary arterial blood, which has gas partial pressures of $P_{pa}O_2$, $P_{pa}CO_2$, and $P_{pa}N_2$. The partial pressures of the gases in the lung unit (P_AO_2, P_ACO_2, and P_AN_2) need to be found. This is another root finding problem, for a set of non-linear functions, that is solved with the false position method.

Initial values of P_AO_2 and P_ACO_2 are trialled.
P_AN_2 is calculated by $P_AN_2 = P_B - P_{H_2O} - P_AO_2 - P_ACO_2$, where P_B is barometric pressure of 760 mm Hg and P_{H_2O} is the saturated vapour pressure of water at 37°C. As diffusion equilibrium is assumed, the partial pressures in the gas phase of the lung unit are the same as those in the blood leaving the lung unit. They are used to calculate the contents of O_2 and CO_2 in the blood leaving the lung unit (C_AO_2 and C_ACO_2) with Kelman's subroutines. C_AO_2 and C_ACO_2 are then calculated from the same trial values of P_AO_2, P_ACO_2, using conservation of mass principles. The contents calculated by these two methods are compared. If they are the same to within acceptable tolerances, the trial P_AO_2 and P_ACO_2 are accepted as the solution. If not, they are used to generate new trial values, and the iterative process continues.

To calculate C_AO_2 and C_ACO_2 using conservation of mass principles (see below), a similar approach to that of West and Wagner was used. As these equations are only valid with F_IO_2 < 1, the limit as F_IO_2 tends to 1 was examined. Starting with F_IO_2 of 0.9999999, the limit as $F_IO_2 \to 1$ was approached in a series of steps. Each step towards the limit reduced the F_IN_2 by a factor of 10, and solution was accepted when two successive steps produced the same P_AO_2, P_ACO_2, and P_AN_2, within acceptable tolerances.

Oxygenator compartment

The oxygenator is modelled as a single lung unit, with set expired minute ventilation $\dot{V}_{OXY}$, set perfusion $\dot{Q}_{OXY}$, and set $F_{OXY}O_2$. It is perfused by blood which has gas partial pressures of $P_{PREOXY}O_2$, $P_{PREOXY}CO_2$, and $P_{PREOXY}N_2$. The partial pressures of the gases in the oxygenator unit ($P_{POSTOXY}O_2$, $P_{POSTOXY}CO_2$, and $P_{POSTOXY}N_2$) need to be found. This root finding problem, for a set of non-linear functions, is identical to that of finding the partial pressures of the gases in the lung unit and is solved using exactly the same method. Indeed, the program calls exactly the same function, but substitutes the input parameters F_IO_2 with $F_{OXY}O_2$, $\dot{V}_A$ with $\dot{V}_{OXY}$, $\dot{Q}_L$ with $\dot{Q}_{OXY}$, $C_{pa}O_2$ with $C_{PREOXY}O_2$, $C_{pa}CO_2$ with $C_{PREOXY}CO_2$, and $P_{pa}N_2$ with $P_{PREOXY}N_2$, and the output parameters of the function are passed to $P_{POSTOXY}O_2$, $P_{POSTOXY}CO_2$, and $P_{POSTOXY}N_2$, instead of P_AO_2, P_ACO_2, and P_AN_2.

Equations used for conservation of mass calculations.

The equations given here are for the lung, but the equations for the oxygenator are identical, but with the substitutions given in the section above entitled "Oxygenator compartment".

Equations used to calculate C_AO_2 and C_ACO_2 using conservation of mass principles. These are the same as used in West and Wagner's program, when N_2 exchange is incorporated. It is assumed that F_ICO_2 = 0. The equations are not valid for F_IO_2 of 1, hence the use of the limit as $F_IO_2 \rightarrow 1$.

F_IO_2 is known, and we are using trial P_AO_2 and P_ACO_2. It was assumed P_B = 760 mm Hg, P_{H_2O} = 47 mm Hg.

Equation A $\quad P_IO_2 = F_IO_2(P_B - P_{H_2O})$

Equation B $\quad P_IN_2 = (P_B - P_{H_2O}) - P_IO_2$

Equation C $\quad P_AN_2 = (P_B - P_{H_2O}) - P_AO_2 - P_ACO_2$

Equation D $\quad C_AO_2 = C_{pa}O_2 + \dfrac{P_IO_2\left[\frac{\dot{V}_A}{\dot{Q}_L}\frac{P_AN_2}{P_IN_2} + 8.63\ \alpha_{N_2}\frac{(P_AN_2 - P_{pa}N_2)}{P_IN_2}\right] - \frac{\dot{V}_A}{\dot{Q}_L}P_AO_2}{8.63}$

Equation E $\quad C_ACO_2 = C_{pa}CO_2 - \left[\dfrac{P_ACO_2}{8.63}\ \dfrac{\dot{V}_A}{\dot{Q}_L}\right]$

<u>The derivation of equation D and E is as follows</u>

The 3 basic conservation of mass equations are

Equation 1 $\quad \dfrac{\dot{V}_{AI}\,P_IO_2 - \dot{V}_A\,P_AO_2}{8.63} = \dot{Q}_L(C_AO_2 - C_{pa}O_2)$

Equation 2 $\quad \dfrac{\dot{V}_{AI}\,P_ICO_2 - \dot{V}_A\,P_ACO_2}{8.63} = \dot{Q}_L(C_ACO_2 - C_{pa}CO_2)$

Equation 3 $\quad \dfrac{\dot{V}_{AI}\,P_IN_2 - \dot{V}_A\,P_AN_2}{8.63} = \dot{Q}_L(C_AN_2 - C_{pa}N_2) = \dot{Q}_L\,\alpha_{N_2}(P_AN_2 - P_{pa}N_2)$

Where

- $\dot{V}_{AI}$ is the inspired alveolar ventilation in l/min BTPS
- The factor of 8.63 is to convert from mL STPD to l BTPS and from content/dl to content /l

- α is the solubility in ml STPD dl^{-1} of blood at 37°C. α for N_2 is 0.0017

Rearranging equation 3

Equation 4
$$\dot{V}_{AI} = \frac{8.63\ \dot{Q}_L\ \alpha_{N_2}(P_A N_2 - P_{pa} N_2) + \dot{V}_A\ P_A N_2}{P_I N_2}$$

$\dot{V}_{AI}$ Rearranging Equation 1

Equation 5
$$C_A O_2 = C_{pa} O_2 + \frac{\dot{V}_{AI}\ P_I O_2 - \dot{V}_A\ P_A O_2}{8.63\ \dot{Q}_L}$$

Substituting $\dot{V}_{AI}$ from equation 4 into equation 5

$$C_A O_2 = C_{pa} O_2 + \frac{P_I O_2 \left[\frac{8.63\ \dot{Q}_L\ \alpha_{N_2}(P_A N_2 - P_{pa} N_2) + \dot{V}_A\ P_A N_2}{P_I N_2}\right] - \dot{V}_A\ P_A O_2}{8.63\ \dot{Q}_L}$$

Which can be rearranged to Equation D used in the program for the conservation of mass calculations

Equation D
$$C_A O_2 = C_{pa} O_2 + \frac{P_I O_2 \left[\frac{\dot{V}_A}{\dot{Q}_L}\frac{P_A N_2}{P_I N_2} + 8.63\ \alpha_{N_2}\frac{(P_A N_2 - P_{pa} N_2)}{P_I N_2}\right] - \frac{\dot{V}_A}{\dot{Q}_L} P_A O_2}{8.63}$$

Assuming $P_I CO_2$ = 0, Equation 2 simplifies to

$$\frac{-\dot{V}_A\ P_A CO_2}{8.63} = \dot{Q}_L (C_A CO_2 - C_{pa} CO_2)$$

Which can be rearranged to give equation E used in the program

Equation E
$$C_A CO_2 = C_{pa} CO_2 - \left[\frac{P_A CO_2}{8.63}\ \frac{\dot{V}_A}{\dot{Q}_L}\right]$$

Equations to calculate the Sweep Gas Flow rate ($\dot{V}_{SWEEP}$)

In the previous section on "Equations used for conservation of mass calculations", the equations for the lung were presented (Equations A – E), with the understanding that with suitable substitutions they could also be used for the oxygenator. Ventilation was expressed as expired ventilation at BTPS.

We are now considering the oxygenator, so these substitutions need to be made. The clinician manipulates the gas flow set on the flowmeter delivering gas to the oxygenator ($\dot{V}_{SWEEP}$), not the somewhat abstract concepts of "expired ventilation of the oxygenator" ($\dot{V}_{OXY}$) and "expired ventilation perfusion ratio of the oxygenator" ($\frac{\dot{V}}{\dot{Q}} oxy$). With a standard ball flowmeter, $\dot{V}_{SWEEP}$ is measured at ATPD.

When it is applied to the oxygenator instead of the lung, Equation 1 can be expressed as

`

$$\frac{\dot{V}_{AI\,OXY} \times F_{OXY}O_2 \times (P_B - P_{H_2O}) - \dot{V}_{OXY} \times P_{POSTOXY}O_2}{8.63} = \dot{Q}_{OXY}(C_{POSTOXY}O_2 - \; C_{PREOXY}O_2)$$

Rearranging we get

Equation 7
$$\dot{V}_{AI\,OXY} = \frac{8.63 \times \dot{Q}_{OXY}(C_{POSTOXY}O_2 - C_{PREOXY}O_2) + \dot{V}_{OXY} \times P_{POSTOXY}O_2}{F_{OXY}O_2 \times (P_B - P_{H_2O})}$$

$\dot{V}_{AI\,OXY}$ is the "effective inspired ventilation of the oxygenator", analogous to "inspired alveolar ventilation". It is expressed at l/min BTPS, differs from $\dot{V}_{OXY}$ due to gas exchange across the oxygenator, and does not include dead space ventilation.

In the model we assumed that there was no dead space ventilation in the oxygenator[1]. $\dot{V}_{SWEEP}$ was expressed at l/min ATPD. On this basis

Equation 8
$$\dot{V}_{SWEEP} = \dot{V}_{AI\,oxy} \frac{\left((P_B - P_{H_2O}) \times (T_{AMBIENT})\right)}{(P_B \times T_{BODY})}$$

Body temperature used was 37˚C (310˚K); saturated vapour pressure of water at 37˚C is 47 mm Hg; Barometric pressure used was 760 mm Hg; Ambient temperature used was 24˚C (297˚K). Substituting into Equation 8

Equation 9
$$\dot{V}_{SWEEP} = \dot{V}_{AI\,oxy} \frac{((760 - 47) \times 297)}{(760 \times 310)}$$

Equations 7 and 9 are used by the program to calculate $\dot{V}_{SWEEP}$

---

[1] To incorporate dead space into the oxygenator Equation 8 would be modified to

$$\dot{V}_{SWEEP} = \frac{\dot{V}_{AI\,oxy} \dfrac{\left((P_B - P_{H_2O}) \times (T_{AMBIENT})\right)}{(P_B \times T_{BODY})}}{\left(1 - \dfrac{\dot{V}_D}{\dot{V}_T} oxy\right)}$$

where $\frac{\dot{V}_D}{\dot{V}_T} oxy$ is the ratio of dead space ventilation to total ventilation in the oxygenator.
