# Supplementary material for: A mathematical model of CO2, O2 and N2 exchange during venovenous extracorporeal membrane oxygenation
Source: Intensive Care Med Exp. 2018 Aug 9;6:25. doi: 10.1186/s40635-018-0183-4 (PMC6085277; doi:10.1186/s40635-018-0183-4)
Supplement: Supplementary file 4 — Figure S1. Shows the “Effect on ${S}_{\overline{v}}{O}_2$Sv¯O2 of different approaches to managing ${\dot{V}}_{SWEEP}$V˙SWEEP”. (PDF 77 kb) [file 40635_2018_183_MOESM4_ESM.pdf]

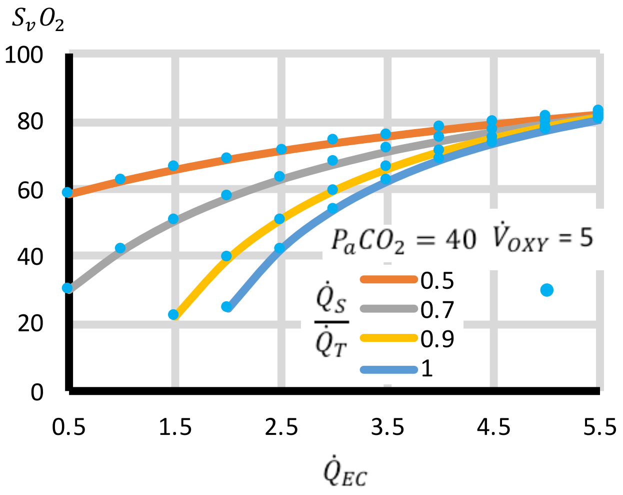

Supplementary Figure 1. Effect on $S_{\bar{v}}O_2$ of different approaches to managing $\dot{V}_{SWEEP}$.

Results for when P_aCO_2 is held constant at 40 mm Hg by varying $\dot{V}_{SWEEP}$ (continuous lines) compared to when $\dot{V}_{SWEEP}$ is held constant at 5 l/min (data points as blue markers). As $\dot{Q}_{EC}$ increases $S_{\bar{v}}O_2$ rises. The higher the $\frac{\dot{Q}_S}{\dot{Q}_T}$, the higher the $\dot{Q}_{EC}$ required to achieve "acceptable" values of $S_{\bar{v}}O_2$ > 60%. *The blue dots of fixed $\dot{V}_{SWEEP}$ = 5 l/min match the curves of variable $\dot{V}_{SWEEP}$ (fixed P_aCO_2), demonstrating that the approach taken to managing $\dot{V}_{SWEEP}$ has minimal effect on $S_{\bar{v}}O_2$.*
