# Supplementary material for: A mathematical model of CO2, O2 and N2 exchange during venovenous extracorporeal membrane oxygenation
Source: Intensive Care Med Exp. 2018 Aug 9;6:25. doi: 10.1186/s40635-018-0183-4 (PMC6085277; doi:10.1186/s40635-018-0183-4)
Supplement: Supplementary file 5 — Figure S2. Shows the “Effect of recirculation at high $\frac{{\dot{Q}}_S}{{\dot{Q}}_T}$Q˙SQ˙T”. (PDF 67 kb) [file 40635_2018_183_MOESM5_ESM.pdf]

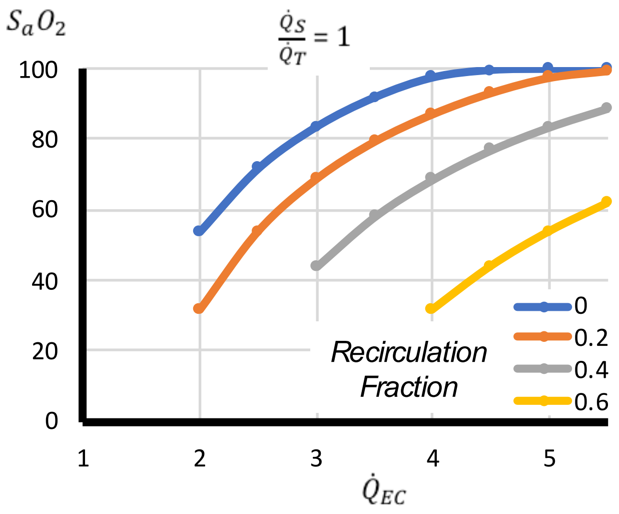


**Supplementary Figure 2. Effect of recirculation at high $\frac{\dot{Q}_S}{\dot{Q}_T}$**

*Results for $\frac{\dot{Q}_S}{\dot{Q}_T}$ = 1. With increasing recirculation there is a large fall in $S_aO_2$, and the scenario becomes untenable unless $\dot{Q}_{EC}$* is high.
