# Supplementary material for: A mathematical model of CO2, O2 and N2 exchange during venovenous extracorporeal membrane oxygenation
Source: Intensive Care Med Exp. 2018 Aug 9;6:25. doi: 10.1186/s40635-018-0183-4 (PMC6085277; doi:10.1186/s40635-018-0183-4)
Supplement: Supplementary file 6 — Figure S3. Shows the “Effect on PaCO2 of Hb”. (PDF 74 kb) [file 40635_2018_183_MOESM6_ESM.pdf]

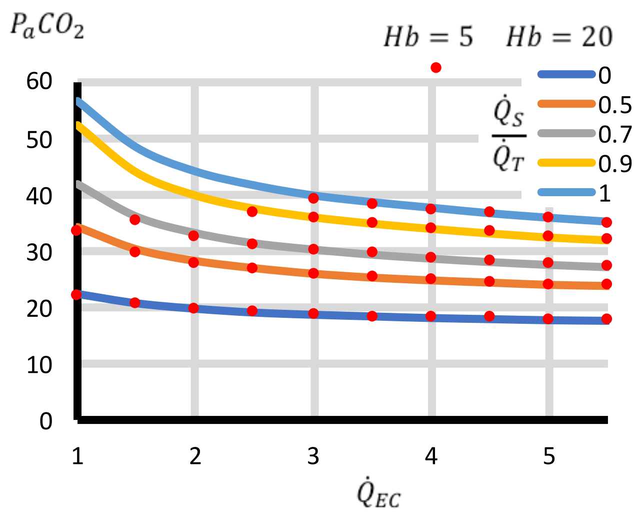

Supplementary Figure 3. Effect on P_aCO_2 of *Hb*.

Lines show the P_aCO_2 for Hb of 20, at each shunt fraction. Red markers show the P_aCO_2 for Hb of 5 at the corresponding shunt fraction. Missing values are for data that is physiologically impossible ($C_{\overline{V}}O_2$ of < 0 would be required), which is more likely when Hb is low, $\frac{\dot{Q}_S}{\dot{Q}_T}$ is high, and $\dot{Q}_{EC}$ is low. At any given value of $\dot{Q}_{EC}$ and shunt fraction, the maximum difference in P_aCO_2 between Hb of 5 and 20 was 1.6 mm Hg, providing both data points were physiologically tenable.
