# Supplementary material for: A mathematical model of CO2, O2 and N2 exchange during venovenous extracorporeal membrane oxygenation
Source: Intensive Care Med Exp. 2018 Aug 9;6:25. doi: 10.1186/s40635-018-0183-4 (PMC6085277; doi:10.1186/s40635-018-0183-4)
Supplement: Supplementary file 7 — Figure S4. Shows the “Effect of FIO2 on PaCO2”. (PDF 74 kb) [file 40635_2018_183_MOESM7_ESM.pdf]

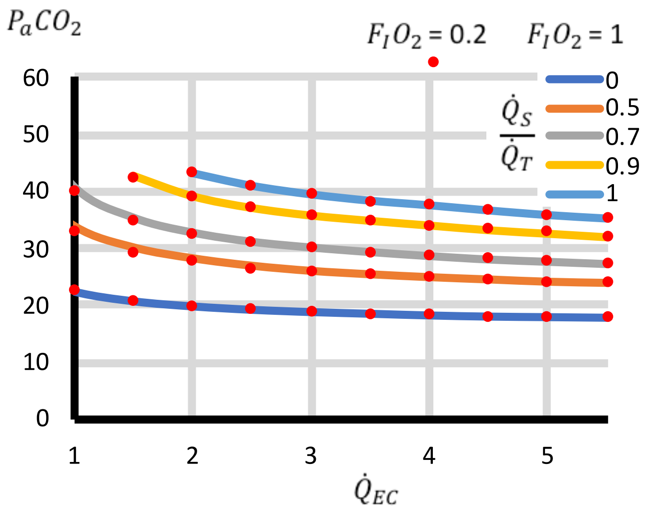

Supplementary Figure 4. Effect of F_IO_2 on P_aCO_2.

Lines show the P_aCO_2 at each shunt fraction, for F_IO_2 of 1. Red markers show the P_aCO_2 at the corresponding shunt fraction, for F_IO_2 of 0.2. Missing values are for data that is physiologically impossible ($C_{\bar{V}}O_2$ of < 0 would be required), which is more likely when F_IO_2 is low, $\frac{\dot{Q}_S}{\dot{Q}_T}$ is high, and $\dot{Q}_{EC}$ is low. At any given value of $\dot{Q}_{EC}$ and shunt fraction, the maximum difference in P_aCO_2 between F_IO_2 of 1 and at F_IO_2 of 0.2 was 1.3 mm Hg, providing both data points were physiologically tenable.
