# Supplementary material for: A mathematical model of CO2, O2 and N2 exchange during venovenous extracorporeal membrane oxygenation
Source: Intensive Care Med Exp. 2018 Aug 9;6:25. doi: 10.1186/s40635-018-0183-4 (PMC6085277; doi:10.1186/s40635-018-0183-4)
Supplement: Supplementary file 8 — Figure S5. Shows the “Effect of FOXYO2 on PaCO2”. (PDF 76 kb) [file 40635_2018_183_MOESM8_ESM.pdf]

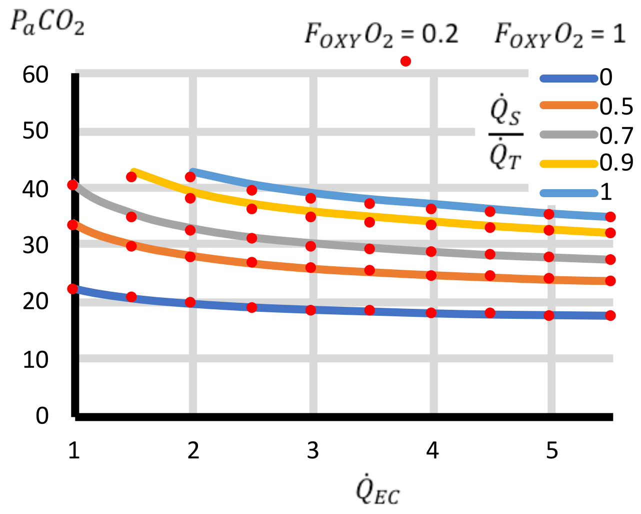

Supplementary Figure 5. Effect of $F_{OXY}O_2$ on P_aCO_2.

Lines show the P_aCO_2 at each shunt fraction, for $F_{OXY}O_2$ of 1. Red markers show the P_aCO_2 at the corresponding shunt fraction, for $F_{OXY}O_2$ of 0.2. At each value of Qec and shunt fraction, P_aCO_2 at $F_{OXY}O_2$ of 1 and at $F_{OXY}O_2$ of 0.2 were within 1.3 mm Hg each other. Missing values are for data that is physiologically impossible ($C_{\bar{v}}O_2$ of < 0 would be required), which is more likely when $F_{OXY}O_2$ is low, $\frac{\dot{Q}_S}{\dot{Q}_T}$ is high, and $\dot{Q}_{EC}$ is low.
