# Supplementary material for: A mathematical model of CO2, O2 and N2 exchange during venovenous extracorporeal membrane oxygenation
Source: Intensive Care Med Exp. 2018 Aug 9;6:25. doi: 10.1186/s40635-018-0183-4 (PMC6085277; doi:10.1186/s40635-018-0183-4)
Supplement: Supplementary file 9 — Figure S6. Shows the “Effect of changes in ${\dot{Q}}_T$Q˙T on PaCO2, over a range of $\frac{{\dot{Q}}_S}{{\dot{Q}}_T}$Q˙SQ˙T”. (PDF 72 kb) [file 40635_2018_183_MOESM9_ESM.pdf]

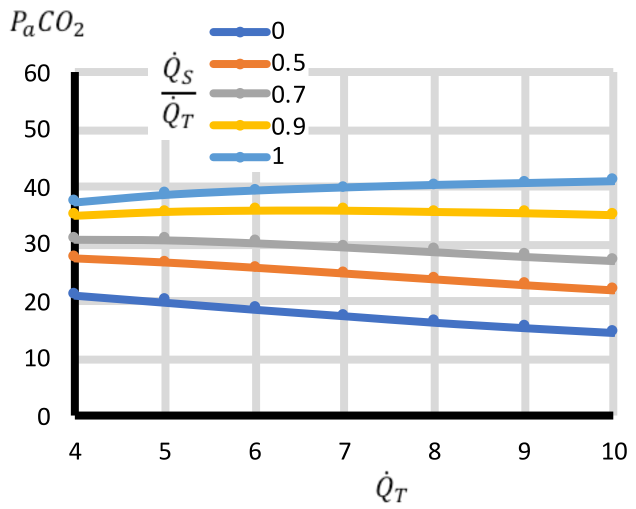

Supplementary Figure 6. Effect of changes in $\dot{Q}_T$ on P_aCO_2, over a range of $\frac{\dot{Q}_S}{\dot{Q}_T}$

Results for $\dot{Q}_{EC}$ = 3 l/min. The effect of increases in $\dot{Q}_T$ depends on the pulmonary shunt fraction. With $\frac{\dot{Q}_S}{\dot{Q}_T}$ of 1, there is a small increase in P_aCO_2 as cardiac output rises; $\frac{\dot{Q}_S}{\dot{Q}_T}$ of 0.9, there is almost no effect. With $\frac{\dot{Q}_S}{\dot{Q}_T} \leq 0.7$, there is a fall in P_aCO_2 as cardiac output rises.
