# Supplementary material for: A mathematical model of CO2, O2 and N2 exchange during venovenous extracorporeal membrane oxygenation
Source: Intensive Care Med Exp. 2018 Aug 9;6:25. doi: 10.1186/s40635-018-0183-4 (PMC6085277; doi:10.1186/s40635-018-0183-4)
Supplement: Supplementary file 10 — Figure S7. Shows the “Sensitivity to which lung model is used”. (PDF 88 kb) [file 40635_2018_183_MOESM10_ESM.pdf]

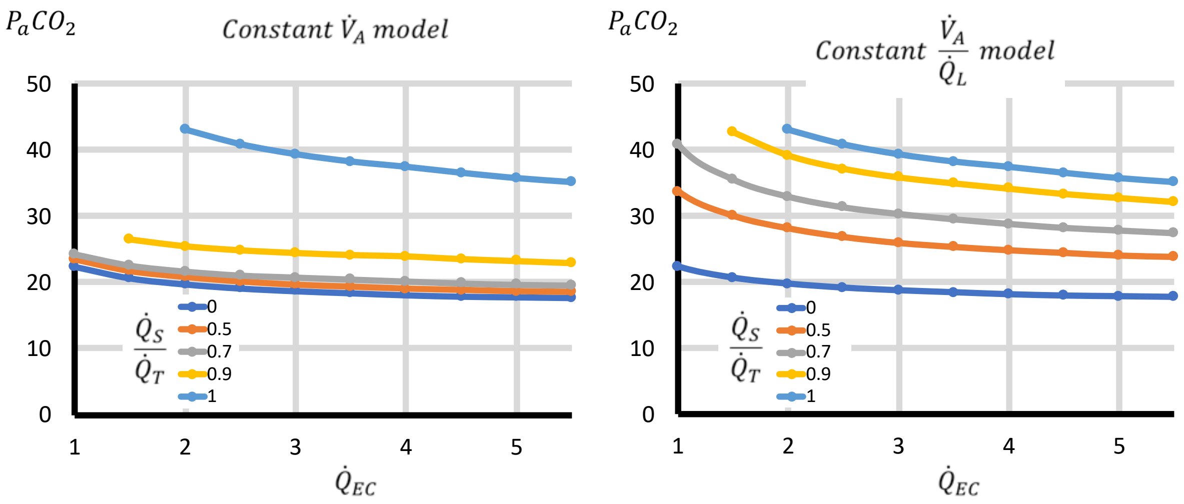

Supplementary Figure 7. Sensitivity to which lung model is used.

$\dot{V}_{SWEEP}$ 5 l/min in both lung models. In the constant $\dot{V}_A$ lung model (left) and the standard ARDS lung model (right) P_aCO_2 rises as $\dot{Q}_{EC}$ falls, but the slope of the line is relatively flat at the $\dot{Q}_{EC}$ > 2L/min. With both lung models P_aCO_2 rises with increasing $\frac{\dot{Q}_S}{\dot{Q}_T}$, but there are differences in the rate of rise between the two models. Curves for $\frac{\dot{Q}_S}{\dot{Q}_T}$ 1.0 are identical in both lung models, as there is no pulmonary blood flow, so $\dot{V}_A$ does not affect gas exchange. Curves for $\frac{\dot{Q}_S}{\dot{Q}_T}$ 0 are identical, as $\dot{V}_A$ is the same in both models.
